# Assessing the frequency and accuracy of morphologic changes of focal bone lesions on [^68^Ga]Ga-PSMA-11 PET/CT in prostate cancer

**DOI:** 10.1007/s00259-025-07331-x

**Published:** 2025-06-17

**Authors:** Thibika Sivakumar, Jakob Heimer, Stephan Beintner-Skawran, Urs J. Muehlematter, Michael Messerli, Noel Spielhofer, Daniel Eberli, Martin W. Huellner, Irene A. Burger, Alexander Maurer

**Affiliations:** 1 Department of Nuclear Medicine, University Hospital Zurich, University of Zurich, Zurich, Switzerland; 2 Department of Nuclear Medicine, Affiliated Hospital for Research and Teaching of the Faculty of Medicine of the University of Zurich, Baden, Switzerland; 3 Department of Mathematics, Seminar for Statistics, ETH Zurich, Zurich, Switzerland; 4 Department of Urology, University Hospital Zurich, University of Zurich, Zurich, Switzerland; 5 Department of HEST, ETH, Zurich, Switzerland

**Keywords:** Unspecific bone uptake, Morphological correlate, Computer tomography, Sclerosis, PSMA-RADS

## Abstract

**Background:**

In [^18^F]F-PSMA-1007 PET imaging, focal bone uptake without morphological correlate (MC) on CT, is often classified as benign. In [^68^Ga]Ga-PSMA-11, intense focal uptake without MC in a classical location is considered suspicious. The prevalence of focal bone uptake with or without MC on [^68^Ga]Ga-PSMA-11 PET/CT remains unclear.

**Methods:**

This single-center, retrospective study included patients who underwent a [^68^Ga]Ga-PSMA-11 PET/CT scan for initial staging or biochemical recurrence (BCR) of prostate cancer between 04/16 − 11/21, with written informed consent for use of clinical data and adequate follow-up. For each patient with focal PSMA accumulation in the bones, up to three of these lesions were scored based on PSMA-RADS 2.0 and a clinical interpretation of suspicion for malignancy including clinical information was provided. In addition, MC on CT were assessed. A composite reference standard including imaging and clinical follow-up data was used.

**Results:**

Out of 824 patients, 323 met eligibility criteria, with 101 showing PSMA-positive bone lesions. 176 lesions were included, 25% of 61 in the staging cohort had no MC, of which 73% were malignant. In the BCR group, 52% of 115 lesions were without MC, of which 48% were malignant. The sensitivity/specificity reached with PSMA-RADS 2.0, and MC on CT was 100%/100%and 78%/36% for staging, and 83%/100% and 60%/72% for BCR, respectively.

**Conclusion:**

Focal [^68^Ga]Ga-PSMA-11 positive lesions without MC on CT are frequent, especially on scans for BCR, with the majority being malignant. Considering PSMA-RADS 4 lesions on [^68^Ga]Ga-PSMA-11 on staging exams, significantly improves the accuracy. Incorporating clinical information and considering PSMA-RADS 3B can further improve the sensitivity for BCR scans.

**Supplementary Information:**

The online version contains supplementary material available at 10.1007/s00259-025-07331-x.

## Introduction

Worldwide, prostate cancer is the second most common cancer in men [1]. Accurate disease staging at initial diagnosis, as well as at early biochemical recurrence (BCR), plays a critical role in determining the most effective treatment approach and prognosis.

The advent of prostate-specific membrane antigen (PSMA) positron emission tomography (PET) imaging has fundamentally changed the landscape of prostate cancer diagnosis and management [2–5]. PSMA PET has been shown to be superior to conventional imaging in large prospective studies [6], with the main advantage of increased sensitivity [6, 7]. False-positive findings on PSMA PET imaging could lead to inappropriate therapy. Therefore, guidelines such as the PSMA Reporting and Data System (PSMA-RADS) have been developed to promote consistent and accurate interpretation [8–10].

A notable complication in PSMA PET interpretation lies in the variability of available radiotracers, with some tracers showing more uptake in non-cancerous tissues [11, 12]. For instance, [^18^F]F-PSMA-1007 frequently demonstrates unspecific bone uptake (UBU) which can complicate image interpretation and lower the specificity [13, 14]. A multicenter analysis found that approximately 51.4% of patients had UBU with [^18^F]F-PSMA-1007 without morphological correlate (MC), a frequency that poses significant challenges for interpretation [13]. Therefore, current recommendations suggest that focal bone uptake without a MC should be considered unspecific when using [^18^F]F-PSMA-1007 to minimize unnecessary follow-up or false-positive diagnoses [13].

On the other hand, focusing only on PSMA positive sclerotic lesions might reduce the sensitivity for bone metastases with tracers such as [^68^Ga]Ga-PSMA-11. However, there is still limited data on how many true positive lesions on [^68^Ga]Ga-PSMA-11 PET/CT show CT correlations. This clinical dilemma does indeed limit the interpretation of focal bone lesions (BL), particularly in distinguishing malignancy from benign uptake, as outlined in a recent editorial [15].

Recent work already confirmed, that PSMA-RADS 4 and 5 lesions on [^68^Ga]Ga-PSMA-11 should be considered suspicious and that PSMA-RADS 3B lesions need careful interpretation including PROMISE guidelines [16]. However, the interpretation of these findings according to a specific clinical setting (staging or BCR) has not been investigated to date. Therefore, we aimed to evaluate the frequency and dignity of BL on [^68^Ga]Ga-PSMA-11 PET/CT, with and without MC, in patients referred for initial staging or assessment of early BCR. In addition, we evaluated the inter-rater agreement for BL based on PSMA-RADS 2.0, clinical interpretation or MC.

## Methods

### Study design and population

This retrospective, single-center study included patients who underwent [^68^Ga]Ga-PSMA-11 PET/CT imaging at our institution between April 2016 and November 2021. Patients were eligible for inclusion if they were referred for initial staging or BCR assessment of prostate cancer. Referrals for indications other than staging or BCR were excluded. Staging was defined as the initial diagnostic workup to determine the extent of disease following a confirmed diagnosis of prostate cancer. This process involved assessing the primary tumor, regional lymph node involvement, and potential distant metastases using the TNM classification system. BCR was defined as a detectable or rising PSA level following definitive primary treatment, such as radical prostatectomy or radiotherapy, prior Androgen deprivation therapy (ADT) was not an exclusion criterion, but any other systemic therapy before PSMA PET was excluded, as well as patients referred for clinical evidence of disease progression on systemic treatments or for therapy selection for internal radioligand therapy.

Patient demographic and clinical information was collected, including age, weight, height, PSA level within four weeks prior to the scan, if available, tumor stage based on the TNM classification, International Society of Urological Pathology (ISUP) grade for histological grading, and the presence of bone metastases. The indication for each PSMA PET/CT scan was documented as either staging or BCR.

The study was conducted in accordance with ICH-GCP guidelines and the Declaration of Helsinki. Informed consent for the anonymized use of imaging and clinical data was obtained from all patients. The retrospective analysis protocol was approved by the local ethics committee (KEK 2023 − 00812).

### Imaging protocols

PSMA-PET/CT was performed using a Discovery MI scanner (GE Healthcare, Waukesha, WI), Discovery 690 Standard scanner (GE Healthcare), Discovery VCT scanner (GE Healthcare) or Discovery ST scanner (GE Healthcare). The injected dose was 2–3 MBq/kg for [^68^Ga]Ga-PSMA-11. The maximum injected dose was not more than 350 MBq. The uptake time was 60 min.

### Image analysis

Image analysis was performed by two nuclear medicine physicians. For each patient, one reader initially selected up to three focal BL based on PSMA PET, prioritizing the most suspicious findings based on increased PSMA uptake, without taking MC into account. The number was limited to three lesions per patient to reduce the patient bias in the analysis and to increase the likelihood that each lesion evaluated would be clinically relevant. This was done in analogy to our previous work to prevent overrepresentation of lesions from single patients with disseminated disease [13]. Selected lesions were then analyzed quantitatively, including the measurement of the standardized uptake value (SUV_max_ and SUV_mean_) for PSMA uptake, PSMA volume (PSMA_vol_) and the corresponding CT-attenuation, expressed in Hounsfield units (HU), within a volume of interest excluding cortical bone.

The selected lesions were then independently evaluated by two readers to determine whether a MC was visible on CT imaging and to assess the likelihood of malignancy: Each lesion was first classified according to PSMA-RADS 2.0 (Table 1) [17]. The presence of sclerotic or lytic changes was considered as a MC. Readers were blinded to the initial imaging reports. In a second step both readers were asked to consider anatomic location, lesion morphology and clinical parameters (PSA level, ISUP grade, patient age, and initial TNM stage) to assess whether lesions should be considered suspicious or non-suspicious for malignancy, resulting in a clinical interpretation (CI).

**Table 1:** PSMA-RADS 2.0 classification [17]

PSMA-RADS 1 (benign)	Benign lesion characterized by biopsy or pathognomonic finding on anatomic imaging and with focal radiotracer uptake.
PSMA-RADS 2 (likely benign)	Equivocal (focal, but low level such as blood pool) uptake in soft tissue site atypical of PCa involvement (e.g., axillary or hilar lymph nodes); equivocal uptake in bone lesion atypical of PCa involvement (e.g., uptake fused to bone lesion and strongly suspected of being degenerative or another benign etiology).
PSMA-RADS 3 (equivocal)	
3A	Equivocal uptake in soft tissue site typical of PCa involvement (e.g., pelvic or retroperitoneal lymph nodes).
3B	Equivocal uptake in bone lesion not definitive but also typical of PCa on anatomic imaging (e.g., pure marrow-based lesion with little if any surrounding bony reaction, lytic or infiltrative lesion, or classic osteoblastic lesion).
3C	Intense uptake in site highly atypical of all but advanced stages of PCa.
3D	Any lesion on CT that requires further workup but does not show any tracer uptake.
PSMA-RADS 4 (PCa highly likely)	Intense uptake in site typical of PCa but lacking definitive findings on conventional imaging.
PSMA-RADS 5 (PCa almost certain)	Intense uptake in site typical of PCa and having corresponding findings on conventional imaging.

*Abbreviations* PCa, prostate cancer

For all lesions classified as PSMA-RADS 3 or 4, follow-up assessments were collected based on previous imaging or additional imaging after implementation of therapy, such as PET/CT, PET/MRI, CT, MRI, bone scintigraphy, or laboratory follow-up with or without therapy. These follow-up assessments contributed to the final benign/malignant classification based on the composite reference standard, in concordance with previous work [16]. In contrast, lesions classified as PSMA-RADS 2 or 5 were not subject to further follow-up, as they are generally regarded as definitively benign or malignant, respectively. This approach reflects standard clinical practice and aligns with the high diagnostic confidence associated with these PSMA-RADS categories.

### Statistical analysis

Continuous variables were summarized as mean and standard deviations (SD) or median and interquartile range (IQR), whereas categorical variables were presented as counts and percentages.

Five different approaches were considered to classify malignancy: Two specialists rating by the PSMA-RADS-2.0 system, the clinical interpretation based on all available clinical parameters and the presence of MC on CT. Inter-rater agreement was assessed using Cohen’s Kappa for binary classifications (clinical interpretation) and Weighted Kappa for ordinal ratings (PSMA-RADS). For these approaches, accuracy, sensitivity, specificity, and the area under the ROC curve (AUC) were calculated for staging and BCR datasets based on the composite reference standard. Confusion matrices were generated to detail true positives, false positives, true negatives, and false negatives. ROCs were plotted to visualize classification performance. For the BCR dataset, the AUC values of the different methods were compared using permutation tests. To adjust for multiple pairwise comparisons, the resulting *p*-values were corrected using the Hochberg procedure.

Differences between the benign and malignant groups were assessed using t-tests or Wilcoxon rank-sum tests for continuous variables and Chi-squared or Fisher’s exact tests for categorical variables, as appropriate.

A *p*-value of less than 0.05 was considered statistically significant.

Statistical analysis was performed with R (version 4.4.1, R Foundation for Statistical Computing). Sankey diagrams were designed with e!Sankey 5.2.1 (ifu Hamburg GmbH, Hamburg, Germany).

## Results

### Patient characteristics

**Fig. 1:**
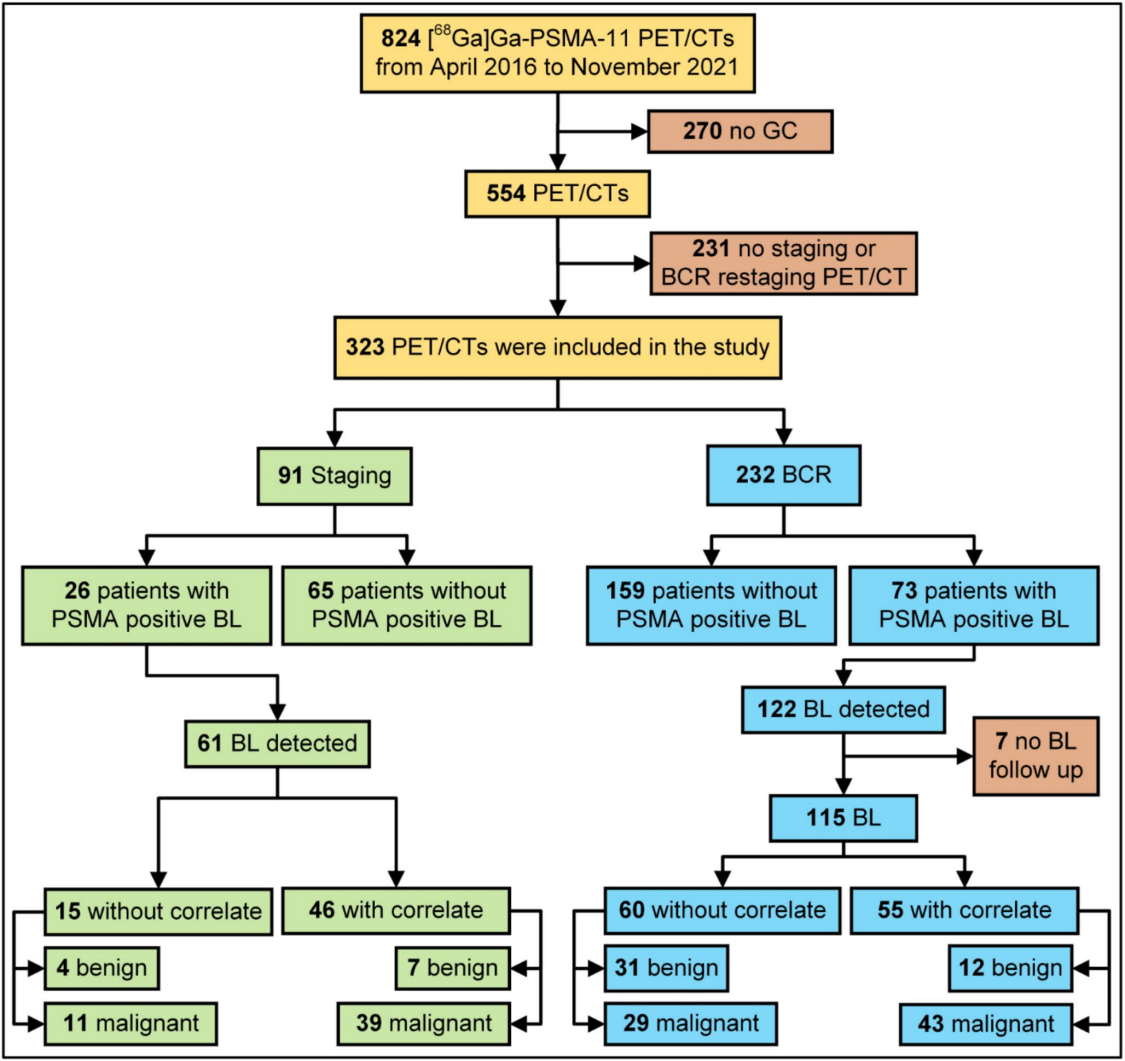
The study flow chart illustrates the number of [^68^Ga]Ga-PET/CTs performed and the number of bone lesions detected. GC, general consent; BCR, biochemical recurrence; BL, bone lesions, PSMA prostate specific membrane antigen (PSMA)

A total of 824 [^68^Ga]Ga-PSMA-11 PET/CT scans were performed between April 2016 and November 2021. Among these, 91 (28%) of the eligible scans were conducted for staging purposes, and 232 (72%) for BCR evaluation (Fig. 1).

Patient characteristics and demographic data for staging are shown in Table 2. 26 of 91 (29%) patients in the staging cohort had detectable BLs. There was no significant difference for age, weight, height for patients with or without PSMA positive BLs. Patients with BL had higher median PSA levels compared to patients without BL with 28.3 ng/ml (IQR: 13.0–75.2), vs. 13.5 ng/ml (IQR: 8.8–29.0), respectively.

**Table 2:** Patient characteristics and demographics for staging

	Staging Cohort	without BL	with BL
Number of patients	91 (28.2%)	65 (71.4%)	26 (28.6%)
Age [y] (median, IQR)	69 (64.5–74.0)	70 (65.0–74.0)	67 (64.0-72.8)
Weight [kg] (median, IQR)	75 (69.0–84.0)	75 (68.0–83.0)	80 (72.0-90.5)
Height [cm] (median, IQR)	173 (168.3-178.8)	173 (168.0-177.0)	173 (170.0-181.5)
Median (IQR) of PSA values [ng/ml]	16.8 (6.9–22.9)	13.5 (8.8–28.8)	28.3 (13.0-75.2)
ISUP grade groups, *n* = 88			
ISUP 1	8 (9.1%)	8 (12.3%)	0
ISUP 2	7 (8.0%)	6 (9.2%)	1 (4.4%)
ISUP 3	16 (18.2%)	12 (18.5%)	4 (17.4%)
ISUP 4	31 (35.2%)	22 (33.9%)	9 (39.1%)
ISUP 5	26 (29.6%)	17 (26.1%)	9 (39.1%)

*Abbreviation* BL, bone lesions

Patient characteristics and demographic data for BCR are shown in Table 3. 73 of the 232 (31%) patients in the BCR cohort, had detectable BL. Patients with and without BL showed no significant differences in age, weight, or height. Unlike the staging cohort, PSA levels were similar between subgroups, with 10.3 ng/ml (IQR: 6.7–20.2) in patients with BL and 10.1 ng/ml (IQR: 6.6–17.7) in those without BL. 9 of 73 (12%) with BCR had ADT before the PSMA PET scan. Final lesion classification according to the composite reference standard identified 50 out of 61 (82%) lesions as malignant in the staging cohort, and 72 out of 115 (63%) lesions as malignant in the BCR cohort.

**Table 3:** Patient characteristics and demographics for BCR

	BCR Cohort	without BL	with BL
Number of patients	232 (71.8%)	159 (68.5%)	73 (31.5%)
Age [y] (median, IQR)	70 (64.8–74.3)	70 (65.0–74.0)	70 (63.5–75.0)
Weight [kg] (median, IQR)	80 (75.0-89.3)	80 (75.0-90.3)	80 (72.0-82.8)
Height [cm] (median, IQR)	175 (170.8-178.3)	175 (172.0-178.5)	172 (169.0-178.0)
Median (IQR) of PSA values [ng/ml]	10.3 (6.6–18.2)	10.1 (6.6–17.7)	10.3 (6.7–20.2)
*Initial T classification*, *n* = 207			
T1	13 (6.3%)	8 (5.7%)	5 (7.5%)
T2	79 (38.2%)	48 (34.3%)	31 (46.3%)
T3	114 (55.1%)	83 (59.3%)	31 (46.3%)
T4	1 (0.5%)	1 (0.7%)	0
*Initial N classification*, *n* = 201			
N0	146 (72.6%)	101 (74.8%)	45 (68.2%)
N1	45 (22.4%)	27 (20.0%)	18 (27.3%)
Nx	10 (5.0%)	7 (5.2%)	3 (4.6%)
*Initial M classification*, *n* = 143			
M0	129 (90.2%)	91 (92.9%)	38 (84.4%)
M1	2 (1.4%)	1 (1.0%)	1 (2.2%)
Mx	12 (8.4%)	6 (6.1%)	6 (13.3%)
*ISUP grade groups*, *n* = 200			
ISUP 1	14 (7.0%)	9 (6.7%)	5 (7.6%)
ISUP 2	38 (19.0%)	25 (18.7%)	13 (19.7%)
ISUP 3	74 (37.0%)	54 (40.3%)	20 (30.3%)
ISUP 4	29 (14.5%)	21 (15.7%)	8 (12.1%)
ISUP 5	45 (22.5%)	25 (18.7%)	20 (30.3%)

*Abbreviations* BCR, biochemical recurrence; BL, bone lesions

The workup that defines the dignity of PSMA-RADS 3 and 4 lesions for the establishment of the composite reference standard and the time to composite reference standard are summarized in the table in Supplement 1.

### Bone lesion with or without correlate on CT

**Fig. 2:**
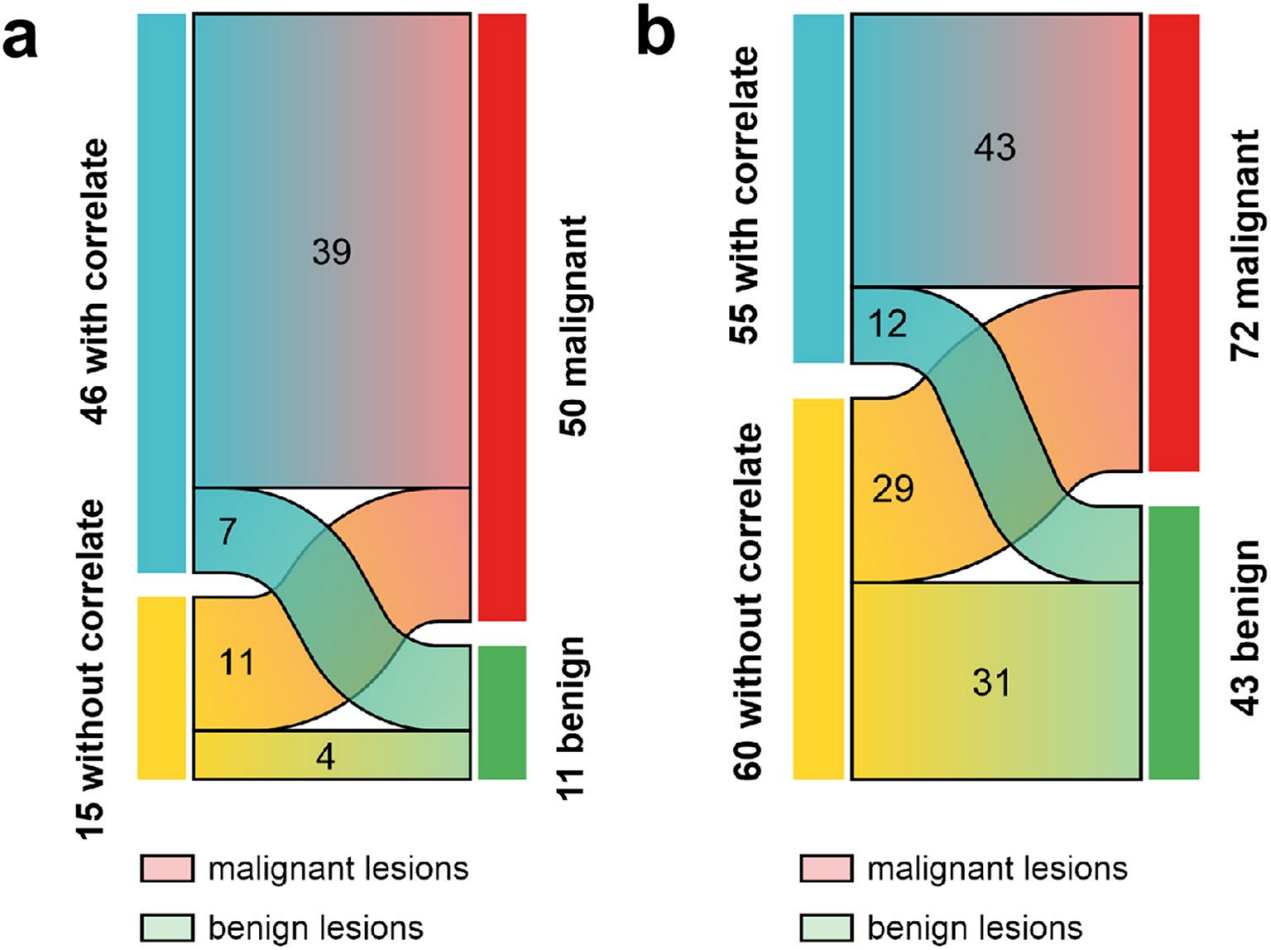
Sankey diagram illustrating the distribution of PSMA-positive bone lesions with or without morphological correlates and the reference standard for true benign and malignant bone lesions (**a**) represents data for primary staging, while (**b**) shows data for biochemical recurrence. The width of each flow is proportional to the number of lesions represented

In the 26 patients referred for staging, a total of 61 BL were selected, of these 25% lacked a MC on CT and 73% without MC were classified as malignant based on the reference standard. Among the 46 lesions with a MC on CT, 15% were categorized benign (Fig. 2a).

In the BCR cohort, 115 BL were analyzed. Here, 52% lacked a MC on CT with 48% classified as malignant based on the reference standard. Among the 55 lesions with a MC on CT, 22% were categorized as benign (Fig. 2b).

### Bone lesion based PSMA-RADS evaluation

**Fig. 3:**
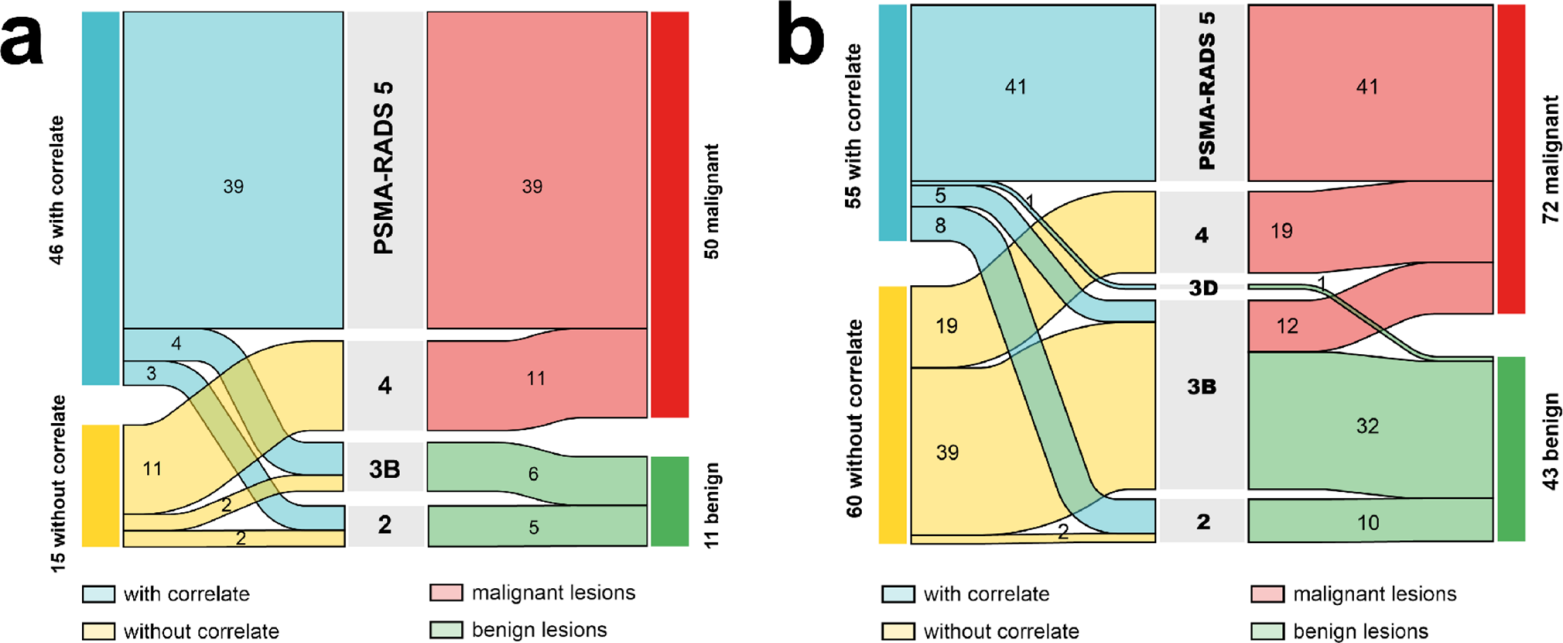
The Sankey diagram illustrates the distribution of PSMA-positive bone lesions with and without morphological correlates, their PSMA-RADS 2.0 classification, and the reference standard for true benign and true malignant bone lesions. (**a**) represents data for primary staging, while (**b**) shows data for biochemical recurrence. The width of each flow is proportional to the number of lesions represented

Based on reader 1, 39 of 46 (85%) BL with a MC on CT were considered PSMA-RADS 5, only 7 BL with MC (15%) were considered unclear or benign (4 PSMA-RADS 3B, 3 PSMA-RADS 2) and all of them were considered benign based on the reference standard. Among 15 lesions without MC, 11 (73%) were classified as PSMA-RADS 4 and confirmed as malignant, while the remaining 4 (27%) were evenly distributed between PSMA-RADS 3B and PSMA-RADS 2, all 4 lesions confirmed as benign (Fig. 3a).

In the BCR cohort, 55 lesions with MC were identified, with 41 (75%) classified as PSMA-RADS 5. The remaining lesions included PSMA-RADS 2 (*n* = 8; 15%), all benign, PSMA-RADS 3B (*n* = 5; 9%), of which 2 were malignant, and PSMA-RADS 3D (*n* = 1; 2%), which was benign. Of the 60 lesions without MC, 19 (32%) were classified as PSMA-RADS 4, all malignant, and 39 (65%) as PSMA-RADS 3B, of which 10 (26%) were malignant. The remaining 2 lesions (3%) were PSMA-RADS 2 and benign (Fig. 3b).

**Fig. 4:**
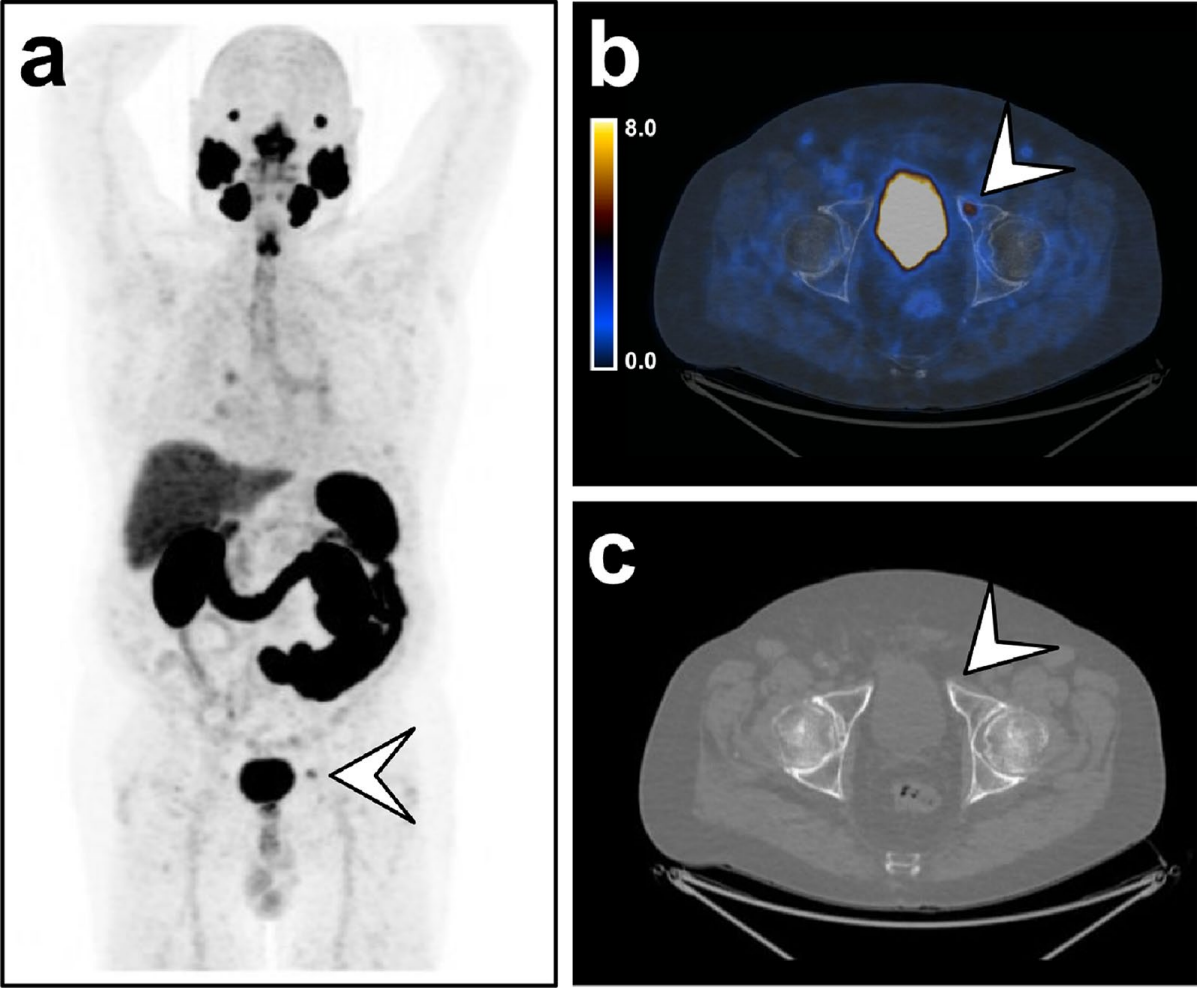
60-year-old male with BCR of PCa underwent [^68^Ga]Ga-PSMA PET/CT for BCR (PSA 3.6 ng/ml) (**a**) Whole-body maximum intensity projection (MIP) image shows a PSMA positive pelvic lesions on the left side (white arrowhead) (**b**) Axial fused [^68^Ga]Ga-PSMA PET/CT shows a lesion in the left anterior acetabular column with a SUV_max_ of 5.3 (white arrow head). (**c**) A CT scan did not reveal any sclerotic morphological correlates (white arrowhead). The lesion was classified as a PSMA-RADS 3B lesion and was rated as malignant. Following radiotherapy of the pelvic lesion, the PSA level normalized and the lesion was confirmed as a true positive bone metastasis

A clinical example of the staging cohort is shown in Fig. 4.

### Bone lesion interpretation including clinical data

**Fig. 5:**
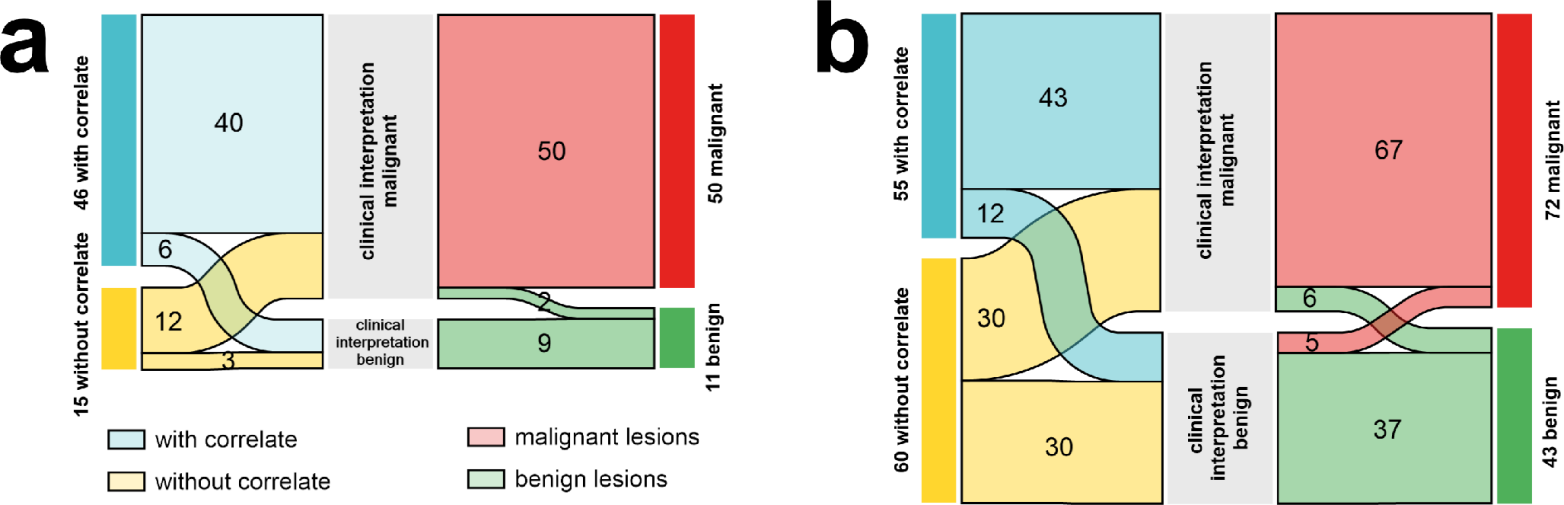
The Sankey diagram illustrates the distribution of PSMA-positive bone lesions with and without morphological correlates, their clinical interpretation as benign or malignant by reader 2, and the reference standard for true benign and true malignant bone lesions. (**a**) represents data for primary staging, while (**b**) shows data for biochemical recurrence. The width of each flow is proportional to the number of lesions represented

In the staging cohort, Reader 1 interpreted of 40 of 46 BL with MC (87%) as malignant, with only 1 misclassification, which was benign according to the reference standard. All 6 benign classifications were accurate. Among 15 BL without MC, 3 (20%) were correctly identified as benign, and 1 of 12 BL (8%) interpreted as malignant was benign according to our reference standard (Fig. 5a).

In the BCR cohort, Reader 1 interpreted 43 of 55 BLs with MC (78%) as malignant, with 1 BL misclassified for both groups. For 60 BL without MC, 30 (50%) were classified as malignant with 4 (13%) misclassified as malignant, and 30 (50%) were considered benign with 5 (17%) misclassifications. Misclassification rates were higher for lesions without MC (15%) compared to those with MC (4%) (Fig. 5b).

### ROC curve for reader I and II (pooled analysis)

**Fig. 6:**
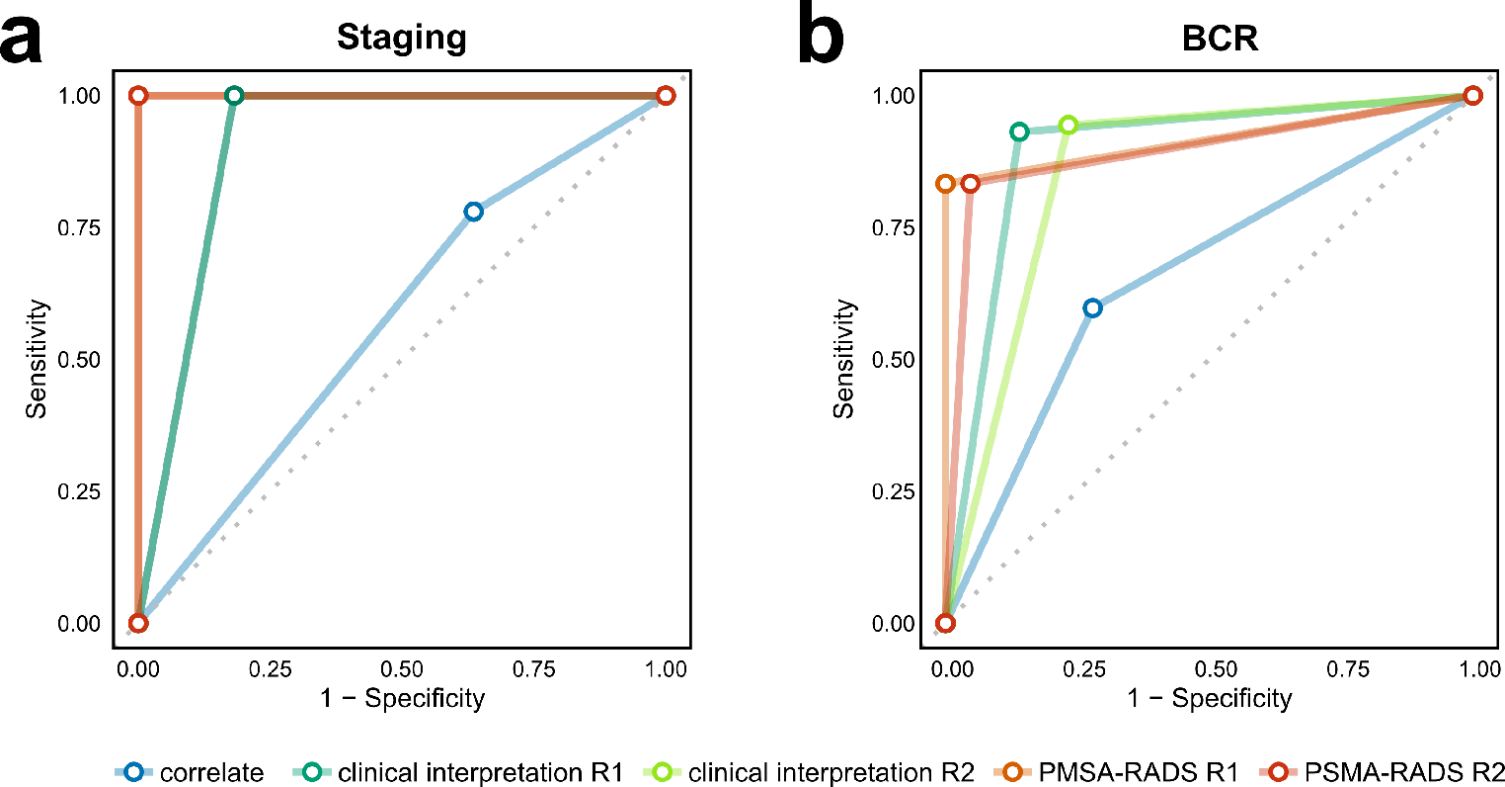
The following five models were analyzed for receiver operating characteristics (ROC): These were correlate, clinical interpretation Reader 1, clinical interpretation Reader 2, PSMA-RADS Reader 1 and PSMA-RADS Reader 2. The analysis encompassed both staging (**a**) and biochemical recurrence (BCR) (**b**)

For the staging assessment, PSMA-RADS classification achieved a perfect diagnostic performance with an accuracy, sensitivity, specificity, and AUC of 1.00. The CI had a slightly lower specificity (0.818), while maintaining high accuracy (0.967) and sensitivity (1.00), resulting in an AUC of 0.909. Simple stratification based on MC on CT exhibited the weakest accuracy of 0.705, with a sensitivity of 0.780 and specificity of 0.364. (Table 4; Fig. 6a).

**Table 4:** Model performance metrics for staging and BCR datasets for both readers

	Accuracy	Sensitivity	Specificity	AUC
Staging				
Clinical interpretation 1	0.967	1.000	0.818	0.909
Reader 1 PSMA-RADS 2.0	1.000	1.000	1.000	1.000
Clinical interpretation 2	0.967	1.000	0.818	0.909
Reader 2 PSMA-RADS 2.0	1.000	1.000	1.000	1.000
Correlate	0.705	0.780	0.364	0.572
BCR				
Clinical interpretation 1	0.904	0.931	0.860	0.896
Reader 1 PSMA-RADS 2.0	0.896	0.833	1.000	0.917
Clinical interpretation 2	0.878	0.944	0.767	0.856
Reader 2 PSMA-RADS 2.0	0.878	0.833	0.953	0.893
Correlate	0.643	0.597	0.721	0.659

*Abbreviations* AUC, area under the curve; BCR, biochemical recurrence

In the BCR assessment, PSMA-RADS classification maintained high specificity but a slightly lower sensitivity (83%), leading to an AUC of 0.917. CI demonstrated high sensitivity but lower specificity with an AUC of 0.896. The BL assessment based on correlate findings on CT exhibited again the lowest diagnostic performance, with an AUC of 0.659 (Table 4; Fig. 6b). Permutation tests demonstrated that the MC model’s AUC was significantly lower (*p* < 0.01) than those based on PSMA-RADS or CI. No statistically significant differences were observed among PSMA-RADS and CI.

### Inter-rater agreement

For staging, Cohen’s Kappa for clinical interpretation showed perfect agreement (κ = 1.000), while Weighted Kappa for PSMA-RADS had near-perfect agreement (κ = 0.983). For BCR, Cohen’s Kappa for clinical interpretation showed substantial agreement (κ = 0.827), and Weighted Kappa for PSMA-RADS had near-perfect agreement (κ = 0.933).

### Correlation with clinical and imaging parameters

**Fig. 7:**
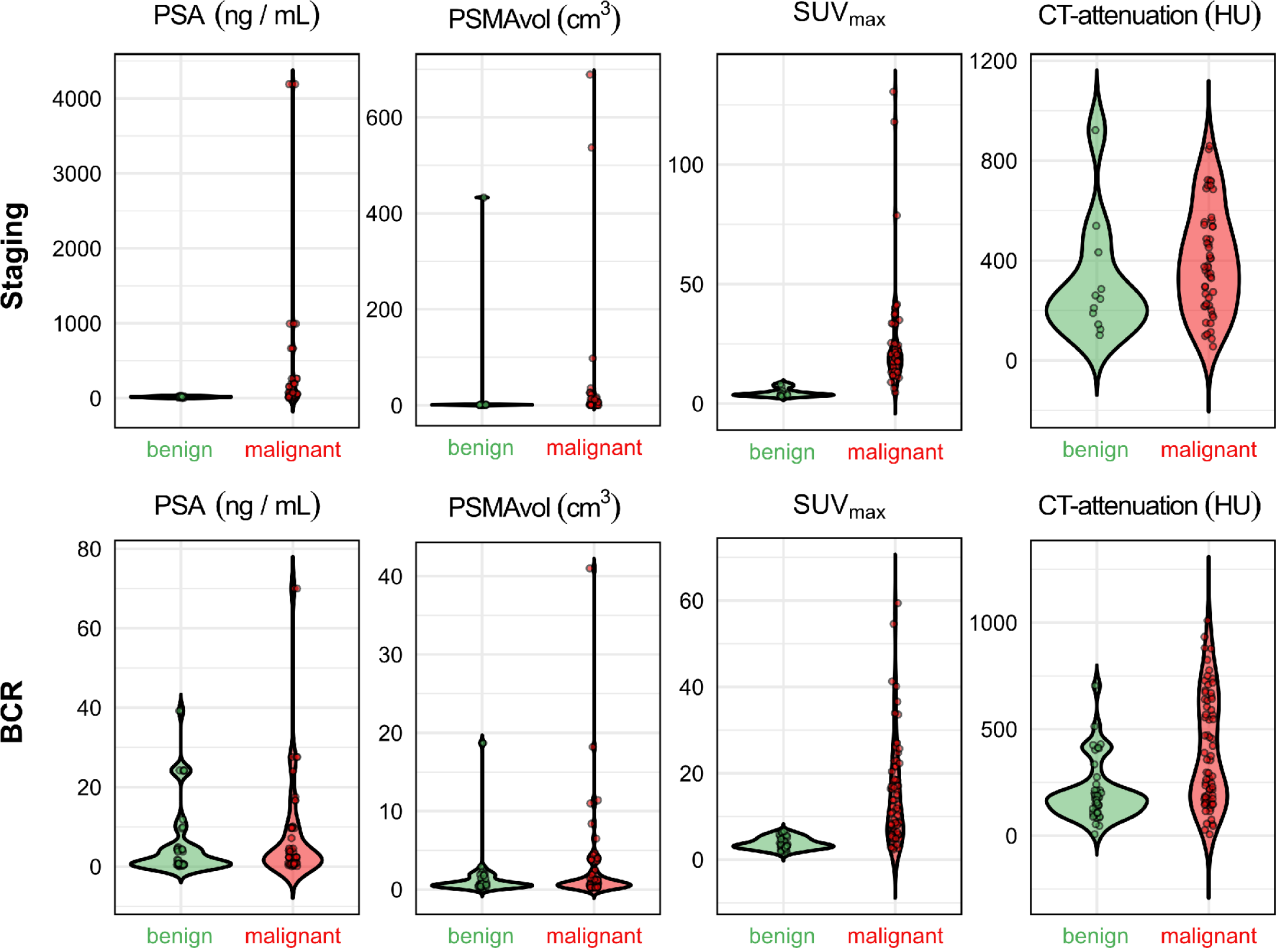
The violin plots illustrate the comparison of PSA, PSMA_vol_, SUV_max_ and CT-attenuation (HU), between benign and malignant cases for both staging (top) and BCR (bottom)

Figure 7 illustrates the distributions of PSA, PSMA_vol_, SUV_max_ and CT-attenuation (HU) between benign and malignant lesions in both the staging and BCR cohorts. For CT-attenuation (HU), the distributions between benign and malignant lesions show significant overlap in both the staging and BCR cohorts. The median values are comparable, and the density profiles indicate that CT-attenuation (HU) does not provide substantial discriminatory power to distinguish benign from malignant lesions. In contrast, PSA shows a clear distinction between the two groups. In both the staging and BCR settings, malignant lesions have a broader and higher distribution of PSA values compared to benign lesions, reflecting the greater variation and higher PSA levels in malignant cases. SUV_max_ shows the most pronounced differences between benign and malignant lesions. In the staging cohort, benign lesions have low SUV_max_ values, whereas malignant lesions have a broader and higher distribution of SUV_max_ values. This pattern is repeated in the BCR cohort. PSMA_vol_ also shows clear differences between the two groups, but with more overlap between the two groups. Finally, ISUP grade 5 and 4 were associated with a higher rate of malignant BL with 84% and 80%, respectively in the staging group, whereas ISUP 3 and 2 were associated with predominantly benign BL (Supplement 2). In the BCR cohort, the pattern was consistent: 86% of BLs in patients with an initial ISUP grade 5 were malignant. Table 5 shows a comparison between the imaging and clinical characteristics of benign and malignant BL. A significant difference is evident in staging for PSA, SUV_mean_, SUV_max_, and in BCR for SUV_mean_, SUV_max_, MC present, CT-attenuation (HU), and ISUP.

**Table 5:** Comparison of various imaging features and clinical parameters of benign and malignant bone lesions

	Benign BL	Malignant BL	*p*-value
Staging			
Number of lesions	11	50	
PSA (median [IQR])	17.8 [9.5, 21.9]	56.0 [28.3, 227.0]	0.001
SUV_mean_ (median [IQR])	2.4 [2.0, 2.9]	11.9 [8.5, 15.2]	< 0.001
SUV_max_ (median [IQR])	3.9 [3.4, 4.8]	19.4 [15.6, 25.3]	< 0.001
PSMA_vol_ (median [IQR])	1.1 [0.8, 1.5]	3.7 [1.0, 11.0]	0.046
MC present (n (%))	7 (63.6)	39 (78.0)	0.539
CT-attenuation (HU) (median [IQR])	247.0 [167.0, 360.0]	374.0 [222.0, 542.3]	0.133
ISUP (n (%))			0.415
2	0 (0)	1 (2.0)	
3	3 (27.3)	5 (10.0)	
4	4 (36.4)	16 (32.0)	
5	4 (36.4)	21 (42.0)	
N/A	0 (0)	7 (14.0)	
BCR			
Number of lesions	43	72	
PSA (median [IQR])	0.8 [0.5, 4.7]	2.3 [0.9, 9.7]	0.134
SUV_mean_ (median [IQR])	2.2 [1.8, 2.8]	6.7 [3.7, 11.3]	< 0.001
SUV_max_ (median [IQR])	3.5 [2.8, 4.7]	10.9 [5.9, 19.1]	< 0.001
PSMA_vol_ (median [IQR])	0.6 [0.4, 1.4]	0.8 [0.4, 1.9]	0.439
MC present (n (%))	12.0 (27.9)	43.0 (59.7)	0.002
CT-attenuation (HU) (median [IQR])	173.0 [111.5, 213.0]	286.5 [171.8, 612.3]	< 0.001
ISUP (n (%))			< 0.001
1	7 (16.3)	3 (4.2)	
2	6 (14.0)	14 (19.4)	
3	19 (44.2)	10 (13.9)	
4	1 (2.3)	7 (9.7)	
5	5 (11.6)	30 (41.7)	
N/A	5 (11.6)	8 (11.1)	

*Abbreviations* BL, bone lesions; HU, Hounsfield Unit; MC, morphological correlate

## Discussion

The evaluation of focal BL in patients with prostate cancer remains a crucial aspect of disease staging and therapeutic decision-making. Accurate differentiation between malignant and benign BL is essential, as it influences treatment strategies and prognostic assessments.

Our results show that 33% of malignant PSMA-positive BL on [^68^Ga]Ga-PSMA-11 PET scans do not have a MC, with a particularly high proportion observed in patients scanned for BCR (40%). This highlights the inadequacy of MC as a prerequisite for identifying metastatic disease. In concordance with Mainta et al. we found a high likelihood of malignancy for PSMA-RADS 2.0 categories 4–5 [16]. Furthermore, they found that PSMA-RADS 3B lesions were malignant in 59% and could be further stratified using PROMISE criteria. However, they did not subdivide the cohort into staging and BCR. Given that 3B lesions are more common in BCR population and less likely to be malignant in patients referred for staging, we believe a more specific interpretation of BL according to the clinical indication is necessary. While for BCR, PSMA-RADS 3B lesions with typical clinical features (high PSA, high ISUP), a more sensitive interpretation may be critical for early detection of recurrence, even in the absence of structural changes. PSMA-RADS 3B lesions should be taken with caution in the staging setting to prevent overstating of patients.

The evaluation based on PSMA-RADS 2.0 demonstrated a high level of inter-reader agreement. The Weighted Kappa for PSMA-RADS classification was extraordinary in both staging (κ = 0.983) and BCR (κ = 0.933), highlighting the reliability of combining structured reporting systems.

Subjective interpretation combining PSMA-RADS 2.0 classification with clinical data slightly increased the inter-reader agreement but still showed a substantial agreement between two experienced readers in the BCR cohort (Cohen’s Kappa = 0.827) and perfect agreement in the staging cohort (Cohen’s Kappa = 1.000). These results are consistent with the high sensitivity and specificity for BL detection reported in the ProPSMA study and further validate the diagnostic accuracy of [^68^Ga]Ga-PSMA-11 PET/CT [6].

In concordance with Mainta et al. with 21% PSMA-RADS 4 lesions of all malignant BL we also found that 22% of malignant BL in the staging cohort were PSMA-RADS 4 lesions and were confirmed malignant based on the composite reference standard. Thus, ignoring lesions that do not correlate on CT will significantly reduce the accuracy of PSMA PET. Higher ISUP grades (4 & 5) were associated with a higher likelihood of malignancy, and there was a strong association between lesion SUV_max_ and malignancy rate, which is also the principle of the PSMA-RADS 2.0 classification [17]. This may also explain why a radiomics score based on PSMA PET/CT incorporating CT and PSMA PET features was able to help unexperienced PET readers to improve their assessment of focal BL [18]. However, a radiomics score did not improve lesion interpretation by experienced readers.

Our study had several limitations. The retrospective, single-center design introduces inherent limitations, including potential selection bias and limited generalizability to broader patient populations or clinical settings. The reliance on a composite reference standard that includes follow-up imaging, and clinical data introduces further potential variability and bias. In addition, follow-up data were not available for PSMA-RADS 5 lesions, which are typically not clinically questioned due to their high likelihood of malignancy. While this reflects routine clinical practice, the lack of follow-up confirmation may limit the ability to definitively validate these lesions, potentially affecting the completeness of the analysis. However, studies prioritizing histopathologic confirmation of BL are very difficult to perform and usually available only in small numbers, with limited information on frequency and prevalence in larger cohorts [19].

## Conclusion

Focal [^68^Ga]Ga-PSMA-11 positive lesions without MC on CT are frequent, especially on scans for BCR, with 52% being malignant. The use of PSMA-RADS 2.0 significantly improves the accuracy of BL classification over interpretation based on MC alone, with excellent inter-rater agreement. Considering PSMA-RADS 4 lesions on [^68^Ga]Ga-PSMA-11 on staging exams, significantly improves the accuracy. Incorporating clinical information and considering PSMA-RADS 3B can further improve the sensitivity for BCR scans.

## Electronic supplementary material

Below is the link to the electronic supplementary material.

Supplementary Material 1

Supplementary Material 2: The Sankey diagram illustrates the distribution of PSMA-positive bone lesions with and without morphological correlates, their ISUP classification groups, and the reference standard for true benign and true malignant bone lesions. (a) represents data for primary staging, while (b) shows data for biochemical recurrence. The width of each flow is proportional to the number of lesions represented.

## Data Availability

The analyzed data may be available from the corresponding author upon reasonable request and with the permission of University Hospital Zurich, University of Zurich, Zurich, Switzerland.
