# Supplementary material for: Assessing the frequency and accuracy of morphologic changes of focal bone lesions on [68Ga]Ga-PSMA-11 PET/CT in prostate cancer
Source: Eur J Nucl Med Mol Imaging. 2025 Jun 17;53(1):231–42. doi: 10.1007/s00259-025-07331-x (PMC12660395; doi:10.1007/s00259-025-07331-x)
Supplement: Supplementary file 1 — Supplementary Material 1 [file 259_2025_7331_MOESM1_ESM.docx]

| Supplementary Table 1: Workup that defines the dignity of PSMA-RADS 3 and 4 lesions for the establishment of the composite reference standard and the time to composite reference standard | | |
| --- | --- | --- |
| Workup | **Number of lesions** | **Time to composite reference standard (days), mean (SD)** |
| Staging | | |
| PET/CT | 8 | 312 (200) |
| PET/MRI | 1 | 636 |
| CT | 3 | 104 (0) |
| PSA level | 5 | 370 (242) |
| BCR | | |
| PET/CT | 30* | 724 (495) |
| PET/MRI | 3 | 440 (172) |
| CT | 5 | 672 (259) |
| MRI | 1 | 42 |
| Bone scintigraphy | 3 | 850 (306) |
| PSA level | 22** | 226 (251) |
| * For a total of three lesions, the time to reference standard could not be calculated due to the lack of an accurate date for the PSA level.  ** For one lesion, the time to reference standard could not be calculated due to the lack of an accurate PET/CT date. | | |
