# Supplementary figures and images for: Assessing the frequency and accuracy of morphologic changes of focal bone lesions on [68Ga]Ga-PSMA-11 PET/CT in prostate cancer
Source: Eur J Nucl Med Mol Imaging. 2025 Jun 17;53(1):231–42. doi: 10.1007/s00259-025-07331-x (PMC12660395; doi:10.1007/s00259-025-07331-x)

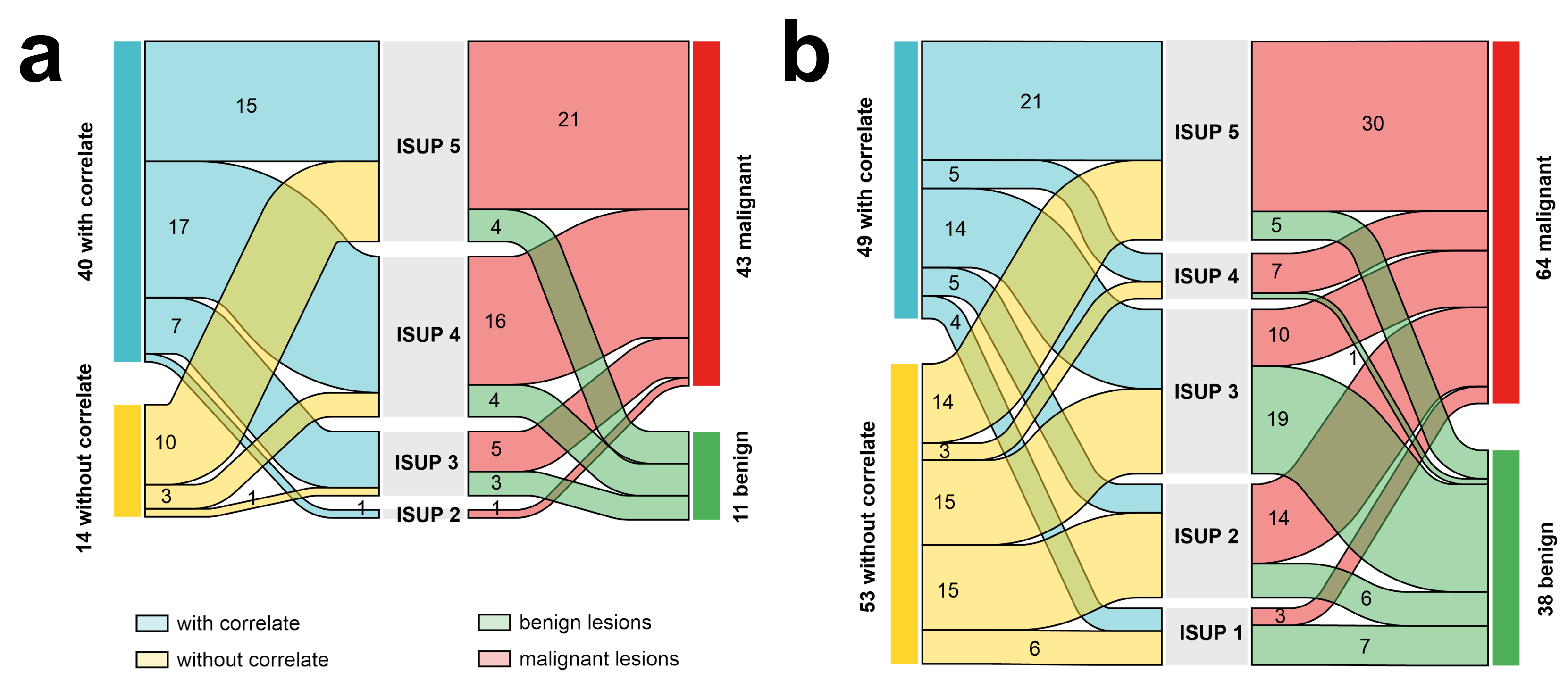

Supplement: Supplementary file 2 — Supplementary Material 2: The Sankey diagram illustrates the distribution of PSMA-positive bone lesions with and without morphological correlates, their ISUP classification groups, and the reference standard for true benign and true malignant bone lesions. (a) represents data for primary staging, while (b) shows data for biochemical recurrence. The width of each flow is proportional to the number of lesions represented. [file 259_2025_7331_MOESM2_ESM.tiff]
